# Experience and limited lighting may affect sleepiness of tunnel workers

**DOI:** 10.1186/1756-0500-7-417

**Published:** 2014-07-03

**Authors:** Dimosthenis Lykouras, Kiriakos Karkoulias, Dimitrios Patouchas, John Lakoumentas, Fotis Sampsonas, Magdalini-Konstantina Tranou, Evanthia Faliagka, Athanasios Tsakalidis, Kostas Spiropoulos

**Affiliations:** 1 Department of Pulmonary Medicine, University of Patras, University Hospital of Patras, Rio, Patras 26500, Greece; 2 Department of Computer Engineering and Informatics, University of Patras, Patras 26500, Greece

**Keywords:** Respiratory, Sleepiness, Shift work, Construction, Tunnel workers

## Abstract

**Background:**

Working on shifts, especially on a night shift, influences the endogenous sleep regulation system leading to diminished sleep time and increased somnolence. We attempted to evaluate the impact of shifts on sleepiness and correlate the sleepiness score to the experience in a shift schedule.

**Materials and methods:**

This cross-sectional study consists of 42 male and 2 female workers involved in a tunnel construction. They underwent spirometry, pulse oximetry and were asked to complete the Epworth Sleepiness Scale questionnaire.

**Results:**

Statistical analysis revealed that workers of lower Epworth had a mean age of 43.6 years, compared to the mean age of 36.4 years of workers with higher Epworth. Furthermore, workers of lower Epworth were characterized by a mean number of shift years equal to 14.8, while those of higher Epworth possessed a mean number of shift years equal to 8. The shift schedule did not reveal any statistically significant correlation.

**Conclusions:**

Workers employed for a longer time had diminished sleepiness. However, there is no relationship between night shifts and sleepiness, possibly because of exposure to artificial lighting in the construction site.

## Background

The modern society has changed towards a 24-h society where the time is no longer a limit for human activity. The unavoidable change in lifestyle and social structure, as well as novel economic and production strategies require shift work to increase production and lower costs [1].

Shift work, especially when night work is needed, dysregulates the relationship between the body’s internal clock and the environment and is associated with shortened sleep, excessive daytime sleepiness, decreased performance and a higher risk for fatal work accidents and road accidents [2,3]. Whether an individual is able to adapt to a working schedule that includes shifts depends on several factors as family status, social and working conditions, personality aspects and coping strategies. The role of each factor is difficult to be evaluated.

The new European development has lead to the adoption of night work by occupational groups that have not used it earlier. Such areas are banking, trading and technical development in order to keep up with strict timetables and complete projects earlier than in the past [4].

An important field where shift working schedules are now greatly used is the motorway construction industry. The aim of motorway worksites is the construction and maintainance of the infrastructure of a motorway such as bridges, tunnels and the road itself. The workers in a worksite can be divided in two categories, those who work on site and are exposed directly to the potentially dangerous construction environment and those who work in the offices at the worksite.

In this study we focus on sleep assessment of tunnel construction workers in a motorway tunnel in Greece by the means of the Epworth Sleepiness Scale. We attempted to evaluate the impact of shifts on sleepiness and correlate the sleepiness score to the experience in a shift schedule.

## Methods

### Subjects

This cross-sectional study consists of 42 male and 2 female workers recruited from the worksite of a large tunnel construction in Greece. The study has been approved by the Ethical Committee of the University Hospital of Patras, Greece. None of the participating workers suffered from any chronic illnesses such as diabetes, arterial hypertension and did not suffer from any respiratory disease.

The shift schedule that was studied involved a day shift from 06.00 to 16.00 and a night shift from 22.00 to 07.00. The workers that were engaged to on-site work performed blasting and drilling of the rock, loading and transport of the blasted rock outside the tunnel, cleaning, rock bolting and cement spraying. The workers outside the tunnel were responsible for the maintenance of the equipment and the cars used in the construction and were mainly mechanics and engineers. The tunnel itself was dark, but the blasting and drilling areas were lit. The construction of a tunnel may demand physical strength and extra effort to cope with.

### Study protocol

Each subject was interviewed by a cooperating doctor on site. We registered data regarding each subject including height and weight in order to calculate BMI, medical history, marital status, smoking habits, coffee consumption, alcohol consumption, prescribed or over-the-counter drugs. Moreover, each patient completed the Epworth Sleepiness Scale and the shift program and experience in shift work were recorded.

### Consent

Written informed consent was obtained from the patients. A copy of the written consent is available for review by the Editor of this Journal.

### Epworth sleepiness scale

The Epworth Sleepiness Scale is a self-completed questionnaire consisted of 8 simple questions attempting to assess the subject daytime sleepiness and its impact on the activity. It has become the world standard method for making this assessment [5].

The ESS asks people to rate the usual chance of falling asleep in 8 different contexts that most people encounter in everyday life. It does not ask people how often they fall asleep in each situation, but only deals with the possibility of falling asleep in the described situation. The total ESS score is the sum of the score in all 8 questions and ranges from 0 to 24. As the total score gets higher, the possibility of daytime sleepiness is increasing as well.

The total ESS score gives an estimation of the average level of sleepiness in everyday life of an individual. The ESS cannot discriminate the factors causing daytime sleepiness, but it is an important tool in defining a person’s sleep-wake health status.

### Statistics

Statistical analysis was performed with SPSS 19.0 (IBM). Testing composite normality was held using the Kolmogorov-Smirnov test. Mean comparisons in parametric continuous data were performed using Student's t-test. Under violation of the normality assumption, median comparisons were performed using the Mann–Whitney U test. Associations between categorical attributes were examined with Pearson's Chi Square test of independence (with Yates' correction for continuity). In all statistical tests, the significance level was defined as p < 0.05, as it is always used in biomedical studies.

## Results

### Demographics and baseline characteristics

A total of 44 workers enrolled in our study. The workers were taken a thorough medical history and were examined for chronic respiratory diseases by the means of lung function testing using spirometry (Pulmolab 435 Morgan Data Acquisition System 401, USA) according to guidelines [6] and pulse oximetry. Only 2 workers reported to have arterial hypertension and were excluded from the study. As it can be seen in Table 1 the spirometry values of the remaining 42 workers were within normal limits.

**Table 1:** Summary of the spirometry values of the workers

**Variable**	**Mean**	**SD**
FEV1 (%)	96,39	11,42
FVC (%)	94,28	10,65
FEV1/FVC	103	10,19
PEFR (l/min)	88,75	18,3
SpO2 (%)	97,69	1,14

The workers had a mean age of 42.3 years (range = 26–58 years) and their working experience in shifts had an average value of 13.7 years. The means and standard deviations of height, weight and BMI are illustrated in Table 2.

**Table 2:** Summary of the continuous characteristics of the workers

**Variable**	**Mean**	**SD**
Age (years)	42,43	8,55
Shift years	13,7	7,75
Height (cm)	174,69	5,43
Weight (kg)	83,05	11,99
BMI (kg/m2)	27,23	3,88

The workers were divided into two main categories: a) tunnel workers who perform inside the tunnels and are involved in drilling of the rock and tunnel construction, and b) worksite workers who are participating in the procedures as technical personnel or even engineers. The group of the tunnel workers had 20 workers, whereas the group of the worksite workers had 22 individuals. In our sample 9 (21%) workers were engaged in the night shift from 22.00 to 7.00 and 33 (79%) worked in the morning shift from 6.00 to 16.00.

As for the family history, 36 workers (86%) were married and 34 of them had at least one child. The remaining 6 workers (14%) were single at the time of the interview.

Nearly all of the workers (93%) drink at least one coffee every day and 69% of them are currently smokers. A number of workers that reaches up to 14 out of the total 42 claim that they consume alcohol on a daily basis, but never during working hours.

### Epworth results and correlation to physiological aspects

We divided the studied population into two groups according to their score in Epworth Sleepiness Scale. The first group contained the subjects that had an Epworth score greater than 10. The second group contained the subjects that had an Epworth score lower than 10. We attempted to identify possible associations of the individual responses to the Epworth Sleepiness Scale against physiological aspects and working features, such as BMI, age, shift years, working post, shift schedule, etc. Statistically significant differences between the compared groups were obtained only in age, shift years, and the shift schedule, among all studied demographic characteristics, the working characteristics, and the spirometry variables. The outcomes of the statistical analysis performed are summarized in Tables 3 and 4.

**Table 3:** Mean comparisons of certain characteristics among the studied groups

**Variable**	**Epworth <10 (n = 35)**		**Epworth >10 (n = 7)**		**Sig. p-values**
	**Mean**	**SD**	**Mean**	**SD**	
Age (years)	43,63	8,3	36,43	7,72	0.04
Shift years	14,84	7,74	8	5,13	0.03
Height (cm)	174,97	5,7	173,29	3,82	NS
Weight (kg)	83,8	11,75	79,29	13,39	NS
BMI (kg/m2)	27,4	3,85	26,37	4,17	NS
FEV1 (%)	96,55	12,12	95,57	7,55	NS
FVC (%)	94,04	10,99	95,5	9,39	NS
FEV1/FVC	103,99	10,05	98,04	10,13	NS
PEFR (l/min)	88,61	18,78	89,46	17	NS
SpO2 (%)	97,69	1,21	97,71	0,76	NS

**Table 4:** Proportion comparisons of certain characteristics among the studied groups

**Variable**	**Epworth <10 (n = 35)**	**Epworth >10 (n = 7)**	**Sig. p-values**
	**n (%)**	**n (%)**	
Post			
*tunnel workers*	14 (40%)	3 (42.86%)	NS
*worksite workers*	21 (60%)	4 (57.14%)	NS
Shift			
*22-7*	5 (14.29%)	4 (57.14%)	0.012
*6-16/16-2*	30 (85.71%)	3 (42.86%)	NS
Marital State			
*children*	29 (82.86%)	5 (71.43%)	NS
*married*	2 (5.71%)	0 (0%)	NS
*single*	4 (11.43%)	2 (28.57%)	NS
Coffee consumption			
*no*	3 (8.57%)	0 (0%)	NS
*sometimes*	22 (62.86%)	7 (100%)	NS
*yes*	10 (28.57%)	0 (0%)	NS
Smoking			
*no*	12 (34.29%)	1 (14.29%)	NS
*yes*	23 (65.71%)	6 (85.71%)	NS
Alcohol consumption			
*no*	14 (40%)	2 (28.57%)	NS
*sometimes*	10 (28.57%)	2 (28.57%)	NS
*yes*	11 (31.43%)	3 (42.86%)	NS

### Epworth and shift work experience

**Figure 1:**
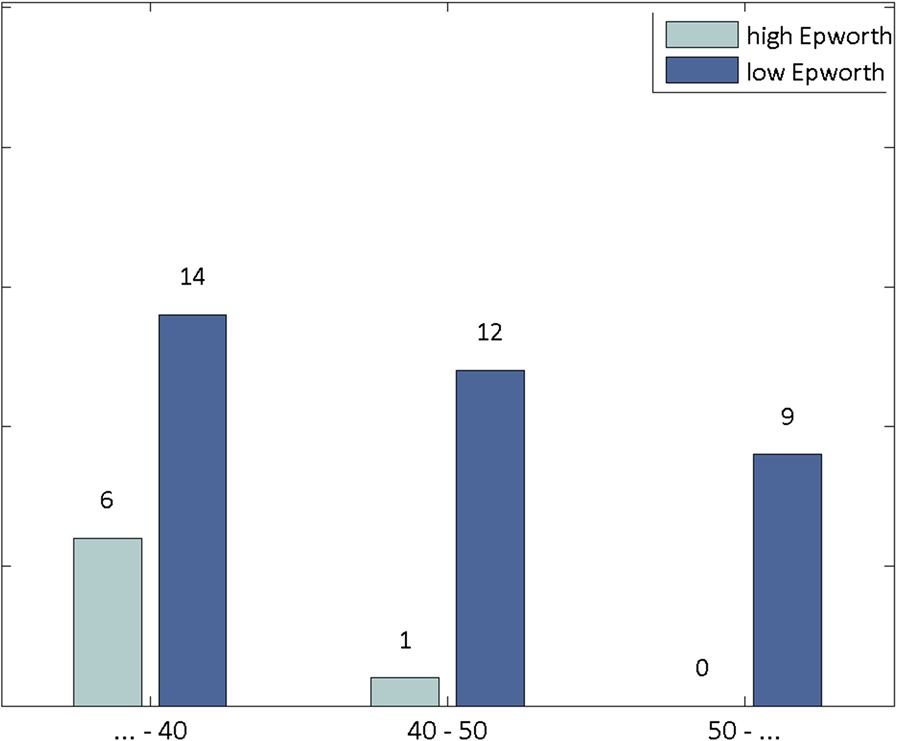
Association between the Epworth Sleepness Scale and age.

**Figure 2:**
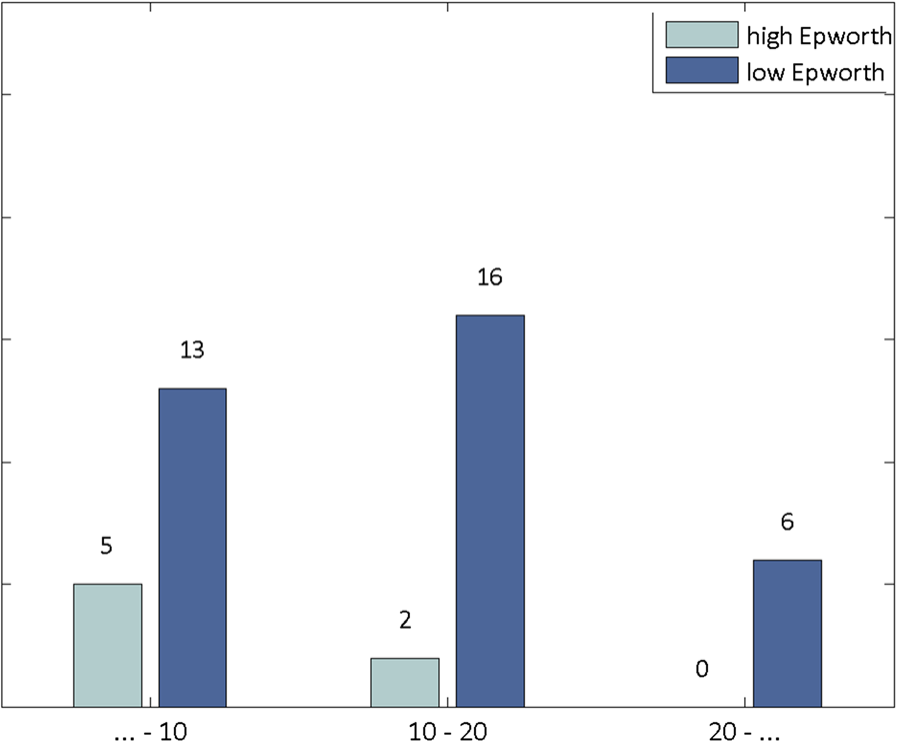
Association between the Epworth Sleepness Scale and shift years.

Statistical analysis revealed that the two groups of subsets differed significantly both in age (Figure 1) and shift years (Figure 2). More specifically, workers of lower Epworth had a mean age of 43.6 years, compared to the mean age of 36.4 years of workers with higher Epworth (t-test, p = 0.04). Furthermore, workers of lower Epworth were characterized by a mean number of shift years equal to 14.8, while those of higher Epworth possessed a mean number of shift years equal to 8 (t-test, p = 0.03). Thus, the group of higher Epworth subjects was comprised of significantly younger and less experienced workers.

### Epworth and shift schedule

A statistically significant association was also identified between the studied groups of workers and their shift schedule. Of the 7 workers with Epworth more than 10, 4 (57.1%) worked at night. In contrast, only 5 of the 35 (14.3%) workers with Epworth less than 10 worked at night (Chi Square test, p = 0.012).

## Discussion

### Shift work and health problems

Shift work results in health problems, because there is a conflict between displaced work hours and the biological clock, which resides in the suprachiasmatic nuclei of the hypothalamus [7][8]. Work at the circadian nadir during night hours will be carried out at low levels of subjective alertness and or physiological activation. The mechanism seems to be light exposure at a particular circadian phase, especially just before the circadian nadir [9,10]. The circadian adjustment of shift workers, however, is influenced both by the environment (e.g. exposure to excessive lighting during night hours) and the exposure to light during the early morning in night shift workers [11]. Thus, adjustment to night work is difficult to be accomplished or at least partial day orientation is maintained.

### Experienced workers are less susceptible to sleepiness

An important finding in our study is that the workers that had been employed for longer time than others had a lower Epworth Sleepiness Scale. Moreover, these workers were older. Thus, there was a doubt if the excessive sleepiness was due to the experience gained or due to their age. As time goes by, the way workers perform their tasks does not remain the same, not only because they change age, but also because their experience gets enriched. Individuals build a kind of self-knowledge, a more or less conscious assessment of their own evolving capacities, faced to work situations.

It is known that experienced workers in dangerous environments (road constructions, heavy industry) want to plan ahead, especially at night. Their main intentions are to limit fatigue and to avoid emergencies [12]. Experience, allows workers to gain familiarity with tasks and acquire the ability to identify critical situations that are likely to occur. The workers gain specific skills and capacities during their professional career, which notably leads them to find the best strategies in order to be efficacious and safe: watch closely the procedures in the worksite and exchange information with the chief. Our analysis supports these previous reports.

### Limited lighting affects sleepiness in shift-workers

In typical individual/worker alertness, performance and metabolism have a peak in the late afternoon and reach a nadir in the early morning. The maximum alertness may interfere with sleep, whereas the nadir is supposed to promote sleep [13]. A night shift worker faces prior sleep termination, in contrast with a day worker.

It is expected that some adjustment of the circadian system across days of night work would ameliorate the negative effects of daytime sleep. However, in our study there seems not to be such a relationship between night shifts and the sleepiness as it is measured by the Epworth Scale. Under optimal conditions, adjustment to a new circadian phase position normally occurs at the speed of 1 h per day, probably through light exposure at the sensitive portions of the circadian phase [14].

Exposure before the nadir would lead to a delay, and after the nadir to an advance. On the other hand, exposure to artificial light during the hours before the circadian trough may remove most of the sleepiness during night work and improve subsequent sleep [15,16]. This might be a possible explanation for our results. Thus, it should be emphasized that there are very few systematic field studies of the application of light in real life work settings.

## Conclusion

In our study it was found that long time exposure of a small group of workers to limited lighting may have an impact on their sleepiness while performing their tasks. Therefore, we think it would be interesting and beneficial to study shift works, more specifically night shifts, and the issues they involve in terms of health and performance at work.

There has been considerable speculation whether long-term shift work would lead to chronic insomnia. However, to date, only a few studies have addressed this issue. However, a few studies have used a retrospective approach [17], and the results seem to suggest a cumulative effect, although the results are not easy to interpret.

Finally, our study attempted to show the correlation between experience in a specific work and sleepiness. Such an experience is valuable especially in night shifts, when the worker is tired, and there are fewer supervisors. This experience can only be gained, however, if the work environment provides an opportunity to make use of it, especially during the night shift, as in the hard working environment of a tunnel construction.

## Competing interests

Authors do not have any conflict of interest.

## Authors’ contributions

DL carried out manuscript preparation and manuscript concept. KK carried out manuscript preparation and manuscript concept. DP carried out field survey. JL carried out statistics. FS carried out manuscript preparation. M-KT carried out statistics. EF mastered data collection. AT drafted the manuscript. KS carried out manuscript concept. All authors have read and approved the final manuscript.
